# Trends in the Japanese National Medical Licensing Examination: Cross-Sectional Study

**DOI:** 10.2196/78214

**Published:** 2025-12-23

**Authors:** Yuki Morimoto, Kiyoshi Shikino, Yukihiro Nomura, Shoichi Ito

**Affiliations:** 1 Department of Medical Engineering Faculty of Engineering Chiba University Chiba Japan; 2 Department of Community-Oriented Medical Education Graduate School of Medicine Chiba University Chiba Japan; 3 Center for Frontier Medical Engineering Chiba University Chiba Japan; 4 Department of Medical Education Graduate School of Medicine Chiba University Chiba Japan

**Keywords:** BERTopic, bidirectional encoder representations from transformers–based topic modeling framework, exam content, Japan, National Medical Licensing Examination, natural language processing, NMLE, taxonomy, topic modeling, trend analysis

## Abstract

**Background:**

The Japanese National Medical Licensing Examination (NMLE) is mandatory for all medical graduates seeking to become licensed physicians in Japan. Given the cultural emphasis on summative assessment, the NMLE has had a significant impact on Japanese medical education. Although the NMLE Content Guidelines have been revised approximately every five years over the last 2 decades, objective literature analyzing how the examination itself has evolved is absent.

**Objective:**

To provide a holistic view of the trends of the actual examination over time, this study used a combined rule-based and data-driven approach. We primarily focused on classifying the items according to the perspectives outlined in the NMLE Content Guidelines, complementing this approach with a natural language processing technique called topic modeling to identify latent topics.

**Methods:**

We collected publicly available NMLE data for 2001-2024. Six examination iterations (2880 items) were manually classified from 3 perspectives (level, content, and taxonomy) based on pre-established rules derived from the guidelines. Temporal trends within each classification were evaluated using the Cochran-Armitage test. Additionally, we conducted topic modeling for all 24 examination iterations (11,540 items) using the bidirectional encoder representations from transformers–based topic modeling framework. Temporal trends were traced using linear regression models of topic frequencies to identify topics growing in prominence.

**Results:**

In the level classification, the proportion of items addressing common or emergent diseases increased from 60% (115/193) to 76% (111/147; *P*<.001). In the content classification, the proportion of items assessing knowledge of pathophysiology decreased from 52% (237/459) to 33% (98/293; *P*<.001), whereas the proportion assessing practical knowledge of primary emergency care increased from 21% (95/459) to 29% (84/293; *P*<.001). In the taxonomy classification, the proportion of items that could be answered solely through simple recall of knowledge decreased from 51% (279/550) to 30% (118/400; *P*<.001), while the proportion assessing advanced analytical skills, such as interpreting and evaluating the meaning of each answer choice according to the given context, increased from 4% (21/550) to 19% (75/400; *P*<.001). Topic modeling identified 25 distinct topics, of which 10 exhibited an increasing trend. Non–organ-specific topics with notable increases included “comprehensive clinical items,” “accountability in medical practice and patients’ rights,” “care, daily living support, and community health care,” and “infection control and safety management in basic clinical procedures.”

**Conclusions:**

This study identified significant shifts in the Japanese NMLE over the past 2 decades, suggesting that Japanese undergraduate medical education is evolving to place greater importance on practical problem-solving abilities than on rote memorization. This study also identified latent topics that showed increased prominence, possibly reflecting underlying social conditions.

## Introduction

### Background

The National Medical Licensing Examination (NMLE) is a mandatory, high-pressure test that all medical school graduates in Japan must pass before becoming licensed physicians. Japan has 82 medical schools with an annual enrollment capacity of more than 9000 students [1]. Upon completing the 6-year medical curriculum, students are eligible to participate in the NMLE program [1]. Unlike licensing examinations in countries such as the United Kingdom, Australia, and Singapore, which primarily assess foreign medical graduates [2], passing the Japanese NMLE is compulsory for all domestic and international graduates who wish to practice medicine in Japan. In 2024, approximately 10,000 candidates took the examination [3]. The NMLE comprises multiple-choice items similar in format to examinations in South Korea, China, Thailand, and Germany [4]. Although there have been discussions regarding transitioning to a computer-based format [1], the examination remains paper-based, with all the candidates in a given year responding to identical items [5]. It is held annually, and all examinees take the examination on the same day. The items and answers are disclosed after the examination, and the item set is completely altered each year. Unlike the United States Medical Licensing Examination (USMLE), repeated attempts are not permitted in the same year, making it a one-shot opportunity. A partially norm-referenced grading system is applied to the pass-or-fail decision, ensuring that approximately 10% of candidates fail each year, thereby maintaining the examination’s competitive nature [1]. Consequently, the pressure on examinees is considerable because failing the examination delays medical licensure by a full year.

Cultural factors contribute to the heightened significance of the NMLE in Japan. The belief that academic achievement determines future stability is deeply rooted in Japanese society, a mindset often associated with Confucian-influenced cultures [1]. This perspective is reinforced by the highly competitive nature of Japanese university entrance examinations, with medical school admissions being particularly competitive and reportedly having an acceptance rate of approximately 8.3% (12 applicants per spot). This fosters a learning culture that prioritizes summative assessments over formative feedback. In this context, students are accustomed to receiving model answers from instructors rather than engaging in critical discussions [6]. These educational values shape how medical students approach learning and preparation for high-stakes examinations such as the NMLE.

The scope and content of the NMLE are defined by the “NMLE Content Guidelines,” published by the Ministry of Health, Labour and Welfare (Japan) [7] (an English summary of which is provided in Multimedia Appendix 1, as no official English version exists). The original Japanese document is titled “*Ishi Kokka Shiken Shutsudai Naiyō Shishin (Reiwa 6 Edition)*.” They specify the fundamental knowledge and skills physicians are expected to possess when taking their first steps into clinical practice. The NMLE Content Guidelines categorize these competencies by topic and outline the performance expectations for each one. To streamline the examination’s administration, the NMLE was reduced from a 3-day, 500-question format to a 2-day, 400-question format [1,7,8]. In addition, the question content was regulated to avoid excessive specialization, and competency levels were clearly defined to ensure clarity regarding the knowledge expected of medical graduates [1]. These guidelines are revised approximately every five years to reflect the evolving requirements for medical professionals.

Another key framework shaping Japanese medical education is the “Medical Education Model Core Curriculum (MCC),” issued by the Ministry of Education, Culture, Sports, Science, and Technology (Japan) [9]. The official Japanese title is “*Igaku Kyouiku Moderu Koa Kyarikyuramu (Reiwa 4 Edition)*.” The MCC guidelines oversee approximately two-thirds of the medical education curriculum and are revised approximately every five years. Since its introduction in 2001, the MCC has evolved significantly; the 2016 revision adopted a competency-based medical education approach and shifted from traditional discipline-based learning to an integrated curriculum [10], aligned with international standards followed in the United States, Singapore, Canada, the Netherlands, and the United Kingdom [11]. The most recent 2022 MCC further elaborates on good practice and offers recommendations for medical education strategy and assessment [12].

Despite these comprehensive educational guidelines, the NMLE continues to exert considerable influence on medical education in Japan. Some universities underemphasize the MCC, and students often prioritize knowledge acquisition for NMLE preparation over more holistic learning experiences [13,14]. To enhance medical education in Japan, it is essential to consider the influence of the NMLE on learning behaviors and curriculum design, particularly given the strong preference for summative assessments in Japanese educational culture. As both the NMLE Content Guidelines and the MCC have evolved, it is increasingly important to examine how the actual content of the NMLE reflects these changes. However, there is a lack of objective longitudinal analysis of the examination itself, highlighting the need for systematic research in this area.

### Study Goal

The objective of this study was to analyze trends in the content of the NMLE across the 21st century. A priori dimensions of interest were defined by the recent NMLE Content Guidelines and included 3 perspectives: level, content, and taxonomy classifications of examination items. Accordingly, the specific research questions were as follows: (1) How has the distribution of examination items changed across these predefined dimensions since 2001? (2) What latent themes, which cannot be fully captured by these dimensions, can be identified through topic modeling, and how have they evolved over time?

Based on prior revisions of the MCC and national examination policy reports, the following a priori hypotheses were formulated: (1) the proportion of items requiring highly specialized knowledge beyond the scope of generalist training has decreased; (2) the overall proportion of practical questions has increased; (3) among the items with higher-order cognitive demands, the proportion of more complex, context-dependent problem-solving items has increased; and (4) in addition to the above dimensions, topic modeling can identify latent themes whose prominence has increased over time, reflecting evolving social priorities and educational expectations. To address these questions, we used a hybrid methodology that integrates rule-based manual classification with exploratory natural language processing (NLP) techniques, particularly topic modeling [15]. Manual classification allowed us to directly reflect the emphases outlined in official medical education guidelines, whereas topic modeling enabled the discovery of previously unknown patterns beyond predefined frameworks.

By integrating these complementary approaches, this study aims to not only provide an evidence-based understanding of how the NMLE has evolved in terms of content emphasis and cognitive skill requirements but also offer practical insights for key stakeholders. For policymakers, our longitudinal data offers insights into the current medical training system’s adaptive flexibility and helps identify remaining areas for further adjustment. For curriculum designers and educators, the trend data serve as an empirical basis for the timely update of educational content to align with contemporary medical education needs. Finally, for medical education researchers, this study’s approach to quantitatively capturing the evolution of examination content offers a new analytical model for education evaluation research, both domestically and internationally.

## Methods

### Study Design

This cross-sectional study comprehensively analyzed NMLE examination items from 2001 to 2024, using a hybrid methodology that integrates rule-based and data-driven approaches in a complementary manner. To elucidate trends in the NMLE, we combined systematic analysis through manual classification with exploratory analysis using artificial intelligence (AI).

In the rule-based component, we focused on 6 specific examination sessions—2001, 2005, 2009, 2013, 2018, and 2024—that each represented the first examination administered after a major revision of the NMLE Content Guidelines. These datasets, comprising a total of 2880 items, were manually classified using 3 classification systems developed based on the guidelines: level, content, and taxonomy.

In the data-driven component, we performed topic modeling across all 24 NMLE sessions conducted between 2001 and 2024, encompassing a total of 11,540 items. This AI-based analysis provided an objective perspective independent of the guidelines, offering valuable complementary insights. Specifically, we used bidirectional encoder representations from transformers–based topic modeling framework (BERTopic) [16,17], a state-of-the-art NLP framework.

### Setting

The NMLE Content Guidelines accompanying the 2024 edition outlined the revisions to be made to the NMLE. The 2024 NMLE was the first to be administered following the revisions [7]. The examination comprises 3 sections: essential, general clinical, and specialized clinical [7]. Each section includes items covering a wide range of medical specialties and public health domains (Figure S1 in Multimedia Appendix 2).

### Classification Procedures

#### Level Classification

The revision process of the NMLE Content Guidelines emphasized the careful selection of diseases to be tested, clarification of the required depth of knowledge for each disease, and the exclusion of topics requiring only basic factual recall that should be mastered before clinical training [5]. To define the breadth and depth of knowledge appropriate for new graduates entering clinical residency, a level classification system was introduced [5]. The 2024 edition of the NMLE Content Guidelines explicitly assigns a level classification to each subcategory within a specialized clinical section [7].

In this study, the level of each question was determined by identifying the disease or condition being tested, either as the main topic or as the correct answer. These were matched to corresponding items in the 2024 NMLE Content Guidelines, and the preassigned level classification was used to categorize each question (Table 1). For items involving multiple key diseases or answer choices, the highest assigned level among the relevant classifications was used to represent the level of the question, with the ranking order as follows: “A > B > C > not classifiable.” Detailed classification criteria are provided in Multimedia Appendix 3.

**Table 1:** Disease categorization. English translations of terms are based on official sources where available. For terms without official translations, translations were made by the authors and validated against published English-language literature.

Level	Disease types	Competencies required for initial treatment	Competencies required for subsequent treatment	Knowledge to be tested
A	Common diseases in primary care settings and acute diseases requiring emergency treatment	Possess enough knowledge to diagnose and manage patients under supervision, while appropriately consulting attending physicians as necessary	Possess enough knowledge to solve problems arising in subsequent treatment	Knowledge of pathophysiology Clinical reasoning skills Knowledge of primary emergency care Knowledge of continued care
B	Diseases that should be learned during postgraduate clinical training	Possess enough fundamental knowledge to manage patients under supervision	Possess enough knowledge to recognize when and how to present concerns to supervisors	Knowledge of pathophysiology Clinical reasoning skills Knowledge of primary care
C	Diseases requiring a high level of clinical experience (beyond the postgraduate clinical training level)	Ability to integrate understanding of the outline of illnesses and clinical reasoning to reach a differential diagnosis	N/A^a^	Ability to recall the names of diseases

^a^N/A: not applicable.

#### Content Classification

The NMLE Content Guidelines impose certain restrictions on test content based on the level classification of each question. As seen in Table 1, the guidelines classify required knowledge such as pathophysiology, clinical reasoning, primary emergency care, and continued care, to help define the focus of examination items [7]. Although explicit definitions of these terms are not provided, they generally align with the cognitive processes expected of a practicing clinician.

This study categorized the items based on the cognitive processes required for clinical practice. The following 4 content categories were defined: pathophysiology, clinical reasoning, primary emergency care, and continued care. Pathophysiology items require reasoning based on a given diagnosis, typically presenting a disease or pathological condition and assessing knowledge of its mechanisms, associated symptoms, and potential complications. Items requiring an approach to making a diagnosis were classified as clinical reasoning. While clinical reasoning is broadly defined and encompasses multiple components—including data gathering, hypothesis generation, problem representation, differential diagnosis, provisional diagnosis, diagnostic justification, management, and treatment [18]—this study applies the term in a narrower sense. Specifically, it is limited to the diagnostic process, as described in the **Accreditation Council for Graduate Medical Education** Internal Medicine Milestones [19], excluding elements related to management and treatment to avoid overlap with other categories. Primary emergency care items focus on urgent decision-making, which is commonly applied in emergency settings and requires immediate problem-solving. Continued care items address long-term management and preventive strategies, often in the context of scheduled outpatient visits or inpatient care. Detailed classification criteria are provided in Multimedia Appendix 4.

Notably, in the official Ministry of Health, Labour and Welfare guidelines, both “primary emergency care” and “continued care” are conventionally categorized under “examination & treatment.” In this study, these were purposefully separated into distinct categories to enable a more process-oriented analysis and to track the evolving focus of the NMLE more accurately.

#### Taxonomy Classification

**Figure 1:**
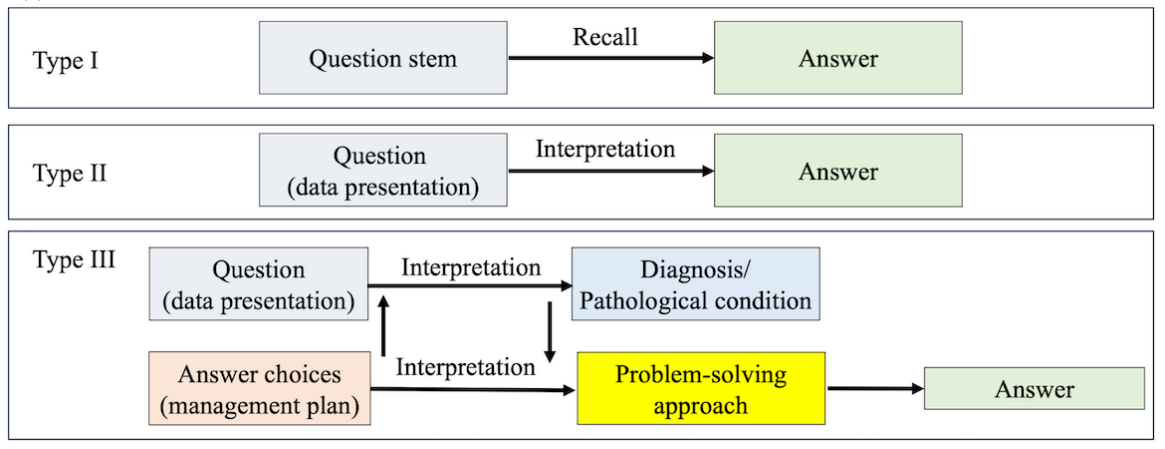
Schematic representation of the cognitive processes required to solve examination questions. Traditional 3-tier taxonomy commonly used in Japan. A potential gap exists between Type II and Type III in terms of cognitive progression.

**Figure 2:**
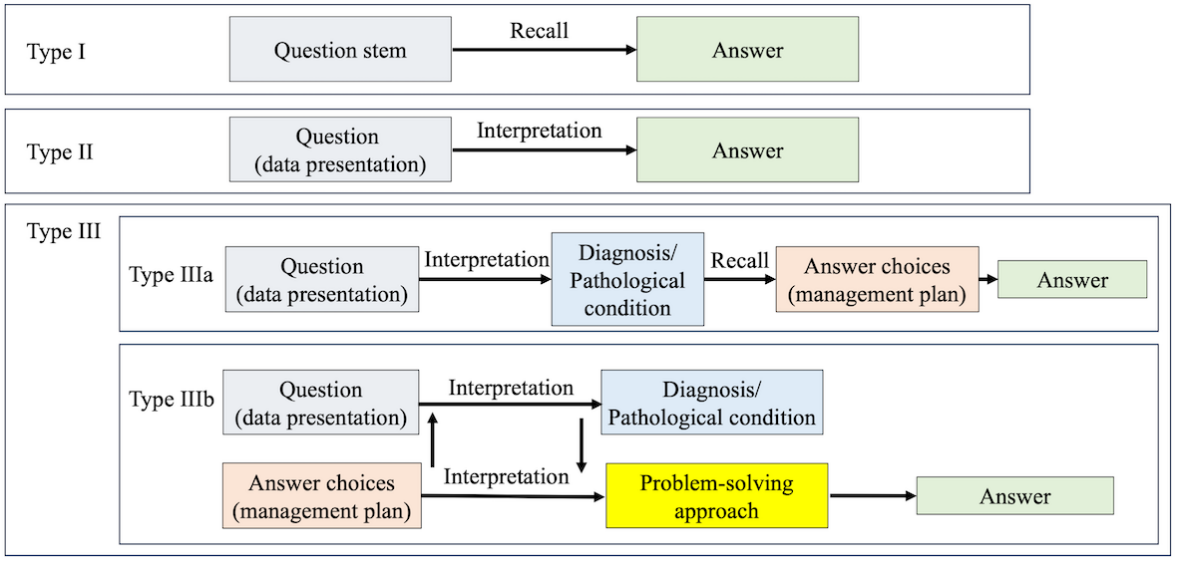
Schematic representation of the cognitive processes required to solve examination questions. Modified 4-tier taxonomy that introduces Type IIIa and Type IIIb to improve continuity between cognitive categories. Type III is defined as a combination of Type IIIa and Type IIIb in this system.

Internationally, examination items in medical education are often evaluated using Bloom’s taxonomy, which categorizes cognitive complexity levels [20,21]. In Japan, a traditional classification system derived from Bloom’s taxonomy is used to categorize items based on the cognitive skills required to answer them. This system classifies items into 3 levels, as outlined in Figures 1 and 2 [5].

- Type I (recall-based items) can be answered purely by recalling factual knowledge.
- Type II (interpretation-based items) require understanding and interpreting the given information to derive an answer.
- Type III (problem-solving items) involve comprehending the scenario, analyzing the meaning of each answer choice, and solving a clinical problem.

Past revisions of the NMLE Content Guidelines have strongly emphasized Type II and Type III items, which test higher-order cognitive skills rather than simple recall.

Although the traditional classification system offers a straightforward framework, it does not guarantee an exhaustive or mutually exclusive categorization of NMLE items. An undefined region between Type II and Type III items may exist. We noted a substantial number of items wherein once the stem was correctly interpreted to reach a diagnostic conclusion, the subsequent answering process required little more than rote recall. Such items often arise when medical students memorize a direct correspondence between diagnoses and their associated treatments. Although prevalent, these items are not adequately defined within conventional classification frameworks.

Therefore, we introduced a novel classification system to address this limitation and enhance exclusivity and comprehensiveness. While retaining the traditional definitions for Types I and II, we refined the definition of Type III and subdivided it into 2 distinct categories: Types IIIa and IIIb. The revised classification system is as follows (Figures 1 and 2):

- Type I (recall-based): Same as the conventional Type I.
- Type II (interpretation-based): Same as the conventional Type II.
- Type III (problem-solving–based): A collective term encompassing Types IIIa and IIIb.
- Type IIIa (interpretation + recall-based): Items in which once the stem is correctly interpreted, the answer can be derived through a simple recall process. This type corresponds to the previously undefined region between Types II and III.
- Type IIIb (interpretation + interpretation-based): Items requiring interpretation of both the stem and the meaning of the answer choices before responding. Unlike Type IIIa, the mere recognition of a diagnosis is insufficient; the examinee must also consider the context and apply higher-order reasoning. This includes scenarios where appropriate decision making is contingent upon factors such as disease severity, stage, comorbidities, allergies, or other patient-specific conditions that influence the selection of an optimal intervention.

The introduction of Type IIIa is particularly relevant, as it addresses a previously unclassified domain wherein interpretation and recall are intertwined. However, the cognitive demand is less rigorous than that in Type IIIb. The distinction between Types IIIa and IIIb is intended to capture the complexity of clinical problem-solving more accurately, particularly in cases where decision making requires contextual understanding beyond simple diagnosis. Detailed classification criteria are provided in Multimedia Appendix 5. To enhance the validity of the revised taxonomy, we aligned the definitions of Types IIIa and IIIb with established educational frameworks, including Revised Bloom’s Taxonomy and the Accreditation Council for Graduate Medical Education Internal Medicine Milestones, thereby ensuring content validity. Furthermore, the distinction between Types IIIa and IIIb reflects whether the clinical reasoning process involves context-sensitive decision making, which supports its construct validity. Reliability was ensured through the use of a detailed classification manual, structured assessor training, calibration exercises, and interrater reliability testing, with discrepancies adjudicated by a senior physician.

#### Topic Modeling

Various topic-modeling techniques exist, such as Non-Negative Matrix Factorization (NMF) and Latent Dirichlet Allocation (LDA). However, with rapid advancements in NLP following the emergence of transformer models, more sophisticated analytical methods have been developed [22]. One such method is BERTopic, an advanced topic-modeling approach that leverages BERT (bidirectional encoder representations from transformers) embeddings, which are context-aware representations of text. Unlike traditional models that assign a fixed meaning to each word, BERT learns word meanings by considering the words that come before and after it. This bidirectional contextual understanding enables the model to capture nuanced relationships between terms—particularly important for medical texts wherein similar concepts can be expressed in diverse ways. By transforming each examination item into a BERT embedding—a numerical vector reflecting its contextual meaning—BERTopic can more accurately cluster semantically related items and identify coherent topics [16]. For Japanese texts, additional preprocessing was required because, unlike English, words are not separated by spaces. We therefore applied morphological analysis using MeCab—the most widely used tool for Japanese language processing—to segment sentences into words. To ensure accurate recognition of medical terminology, a specialized dictionary (MANBYO) was incorporated into MeCab. We then used a Japanese Sentence-BERT model (“sonoisa/sentence-bert-base-ja-mean-tokens-v2”) to generate embeddings, which provided context-aware numerical representations of each examination item (stem + answer choices). These embeddings were subsequently processed through dimensionality reduction and clustering—core steps of the BERTopic topic modeling pipeline. Owing to its high customizability, BERTopic is well suited for applications beyond English-language texts and particularly effective for time-series analyses [23]. It has gained attention for its ability to uncover novel patterns and provide insights into textual data [17,23].

In this study, we applied BERTopic by inputting textual data that combined the item stems and answer choices for each examination item. The topic-modeling process was conducted in six steps using the BERTopic Python package:

- Preprocessing: For the tokenization of the Japanese text, we used the morphological analyzer “MeCab” [24,25]. To ensure appropriate analysis of texts containing symptoms and disease names, we applied a specialized dictionary for medical terms, “MANBYO” [26].
- Embedding: As a Japanese Sentence Transformer model, we used “sonoisa/sentence-bert-base-ja-mean-tokens-v2,” available on Hugging Face [27].
- Dimensionality Reduction: Uniform Manifold Approximation and Projection (UMAP) was used.
- Clustering: Hierarchical Density-Based Spatial Clustering of Applications with Noise (HDBSCAN) was used.
- Tokenization: CountVectorizer was used.
- Weighting Scheme: Class-based term frequency–inverse document frequency (c-TF-IDF) scoring was used.

After analyzing the extracted keywords and various representative items, the authors determined the topic labels through mutual agreement.

To enhance transparency, we also describe the process of parameter selection in BERTopic. While default settings were maintained whenever possible, 3 key parameters were adjusted through systematic trial-and-error to optimize interpretability and stability. For UMAP, n_neighbors was set to 3 to emphasize local structures and uncover fine-grained latent topics, and n_components was set to 10 to preserve information while ensuring clustering stability. For HDBSCAN, min_cluster_size was set to 20, which balanced interpretability and granularity and resulted in 25 coherent topics. We also confirmed that the main conclusions of this study, such as the increase in items under “comprehensive clinical items” and “accountability in healthcare and patient rights,” remained stable across reasonable parameter variations.

Each examination item was assigned to a single topic only, reflecting the multiclass nature of the clustering process. While some items (eg, questions combining “renal disease” and “research”) could plausibly relate to more than one topic, the dimensionality reduction and clustering steps ultimately placed each item in one cluster. This design choice facilitates clearer identification of longitudinal shifts in topic prevalence, whereas a multilabel approach would have assigned multiple simultaneous labels and obscured such temporal trends.

In addition, the process of topic interpretation and labeling was refined. For each topic, the top 20 representative keywords with the highest c-TF-IDF values and the list of examination items assigned to that topic were extracted. The research team carefully reviewed these materials, designating clearly irrelevant words (eg, laboratory units, particles, auxiliary verbs) as stop words to improve the quality of the keyword lists without altering the BERTopic analysis. Final topic labels were established through repeated focus group discussions, with reference to representative items and prior literature, until consensus was reached. For transparency, the representative keyword lists for each topic are provided in Table S2 in Multimedia Appendix 6.

Following the automatic clustering, several closely related topics were manually integrated through consensus discussions (eg, merging “male” and “female” patient clusters into “comprehensive clinical items”) to enhance thematic coherence and prevent over-splitting.

### Selection Criteria for Analysis

For each rule-based analysis, we examined question datasets from the NMLEs for 2001, 2005, 2009, 2013, 2018, and 2024. The 2001 NMLE was included because of its coincidence with the initial publication of the MCC. For the level classification analysis, only items from the specialized clinical section were considered, as the 2024 NMLE Content Guidelines have defined level classifications for this section. Items without an explicitly assigned level were excluded from the statistical analysis. For the content classification analysis, items related to public health knowledge (eg, health policies, legal frameworks, and statistical concepts) were excluded because of their distinct nature. The analysis focused on items from other medical specialties, whereas items that could not be assigned to a predefined content category were excluded from statistical analysis. For the Taxonomy classification analysis, all items were included without exception.

For the data-driven analysis, we used complete question datasets from the 2001-2024 NMLEs. All items were included in the topic-modeling analysis without exception.

Between 2001 and 2006, each examination included 30 or 50 pilot items that were not subject to scoring. However, as the specific pilot items were not disclosed, the dataset was analyzed with all items included.

### Study Variables

The primary outcome variable was the distribution of items across the 3 classification systems in each examination year. The primary predictor variable was the examination year, which was treated as representing time progression, allowing for the assessment of trends in the item distribution. Secondary outcome variables included topic distribution, measured as the proportion of items assigned to each topic, and the slope coefficient from the linear regression, which represented the annual rate of change in the proportion of each topic over time.

### Data Sources and Measurement

Each classification system was evaluated by 10-12 assessors, including clerkship students and licensed physicians. All assessors were provided with a detailed classification manual developed iteratively through expert discussion (Multimedia Appendices 3-5). Prior to the formal classification, assessors participated in structured training sessions, which included video-based instructions and multiple practice rounds with sample items. These sessions were followed by calibration exercises wherein assessors independently classified a pilot set of items, then discussed discrepancies with senior physicians to ensure consistent interpretation of the manual. Clerkship students were included only if they had completed at least one year of clinical clerkship. In addition, when required, their classifications were cross-checked against those of licensed physicians to confirm reliability. The initial draft of the manual was created by a general internist (YM) with reference to the 2024 (Reiwa 6) NMLE Content Guidelines and the official report of the Ministry of Health, Labour and Welfare’s Working Group on Improvements to the NMLE. The draft was reviewed by the study team and refined through expert focus group discussions involving KS, an experienced physician and medical educator. During these discussions, operational rules were established for each system. For the level classification, each item was mapped to the most relevant blueprint subitem, and when multiple disease themes or answer keys were plausible, assessors selected the theme most central to the item’s core; if options were equally plausible, priority was given to the condition with higher frequency or urgency (A > B > C). For the content classification, we adopted 4 mutually exclusive categories—pathophysiology, clinical reasoning, primary emergency care, and continued care—derived from the official guidelines, and refined definitions to avoid overlap. For the Taxonomy classification, we clarified the boundaries among Types II, IIIa, and IIIb through iterative examination of numerous examination items and consensus-based discussions among the research team. In particular, the cognitive distinction between “recall” and “interpretation” was deliberated extensively to define the boundary between Types IIIa and IIIb. Disagreements encountered during manual development were resolved through consensus, supported by additional sample questions when necessary. When discrepancies occurred, a third-party adjudicator (a general internist) made the final decision. We believe that this structured process ensured that all assessors, including students, were adequately trained and able to apply the classification framework reliably.

In this study, clerkship students are defined as medical students who have passed common achievement tests (computer-based tests and objective structured clinical examinations), and been certified to possess the necessary knowledge, skills, and professionalism to participate in clinical clerkships. Given the importance of clinical experience in accurate classification, content and taxonomy classifications were conducted exclusively by licensed physicians or clerkship students with at least two years of clinical clerkship experience.

This ensured that classifications requiring higher clinical expertise were performed only by adequately trained and experienced assessors. All translations of Japanese terms were based on official bilingual documents whenever available. When no official translation was available, translations were performed by the study team and cross-checked against existing English-language literature to ensure consistency and accuracy.

### Statistical Analysis

Interrater reliability of the classification system was assessed using Fleiss’ κ coefficient [28] (0.8-1.0=almost perfect; 0.6-0.8=substantial; 0.4-0.6=moderate; 0.2-0.4=fair). Chi-square tests were conducted to evaluate the relationship between item distribution and examination year. Cochran-Armitage trend tests were conducted to assess time-series trends in question distribution. Statistical analyses were performed using R (version 4.3.3; The R Project for Statistical Computing). The trends of each topic over time were traced using linear regression models based on topic frequencies using NumPy (version 1.26.4; NumPy Developers) in Python (version 3.12.4; Python Software Foundation).

### Ethical Statement

This study did not collect any human or patient information and was thus exempt from ethical approval, informed consent requirements, and institutional review board approval. Additionally, as no identifying information was included, the data did not need to be anonymized or deidentified. No compensation was offered because there were no human participants in this study.

## Results

### Overview

Fleiss’ κ coefficients were calculated to assess the reliability of all 3 classification systems (level, content, and taxonomy). Most coefficients indicated substantial agreement (κ>0.6), although the content classification in the 2013 examination showed a moderate κ coefficient (κ=0.54). Detailed values for each classification across 6 representative examination years are provided in Table S3 in Multimedia Appendix 7, which also describes the procedures for calculating κ coefficients. For the level, content, and taxonomy classifications, a chi-square test revealed a significant association between examination year and the distribution of question categories (*P*=.02, *P*<.001, and *P*<.001, respectively). Based on these results, Cochran-Armitage trend tests were conducted for all 3 classifications to evaluate temporal trends.

### Level Classification

**Figure 3:**
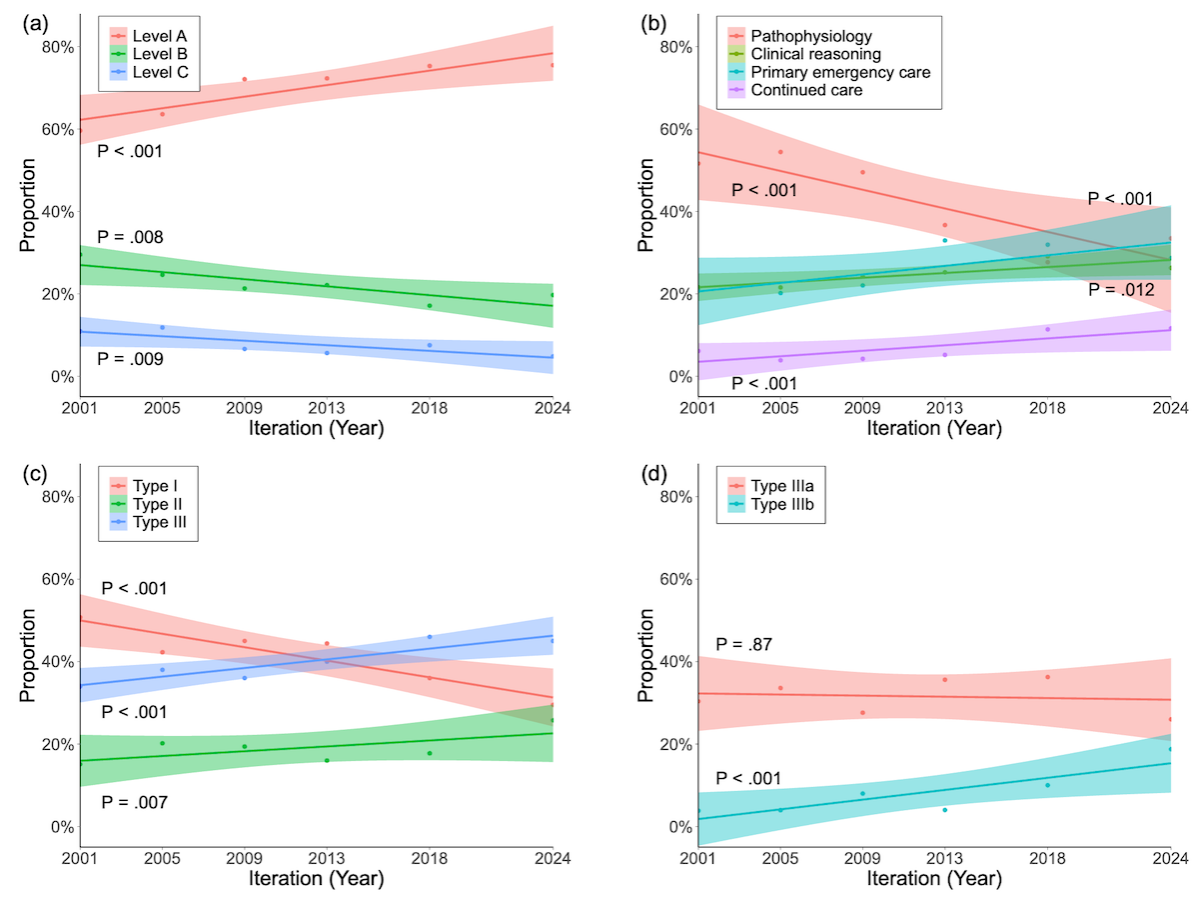
Temporal trends in question distribution across classification systems. (A) Level classification, (B) content classification, and (C) taxonomy classification (Types I, II, and III), and (D) taxonomy classification (Types IIIa and IIIb). Points represent observed data. Solid lines indicate fitted trends estimated using linear regression, with shaded areas representing the 95% CIs. Cochran-Armitage trend tests were performed for each category, and the associated *P* values are displayed. The significant increase in Type IIIb questions observed in subpart D primarily accounts for the overall increase in Type III questions shown in subpart C, whereas no significant trend was found for Type IIIa.

Among the 1100 items in the specialized clinical section, a total of 1073 (97.5%) were successfully classified into one of 3 predefined levels (Table 2). The remaining 27 (2.5%) items were classified as “not classifiable” and excluded from further statistical analysis. Level A items accounted for 743 (69.2%) of classified specialized clinical items, making them the most common. Items at levels B and C accounted for 244 (22.7%) and 86 (8%), respectively. Over 23 years, the proportion of level A items increased significantly from 59.6% (115/193) in 2001 to 75.5% (111/147) in 2024 (*P*<.001). By contrast, the proportion of level B and C items declined significantly, from 29.5% (57/193) to 19.7% (29/147; *P*=.008) and 10.9% (21/193) to 4.8% (7/147; *P*=.009), respectively (Figure 3A). These trends suggest a shift toward assessing higher-priority knowledge over time.

**Table 2:** Level classification.

Year	2001 (n=200), n (%)	2005 (n=200), n (%)	2009 (n=200), n (%)	2013 (n=200), n (%)	2018 (n=150), n (%)	2024 (n=150), n (%)	Total (n=1100), n (%)
Level A	115 (57.5)	124 (62)	142 (71)	141 (70.5)	110 (73.3)	111 (74)	743 (67.6)
Level B	57 (28.5)	48 (24)	42 (21)	43 (21.5)	25 (16.7)	29 (19.3)	244 (22.2)
Level C	21 (10.5)	23 (11.5)	13 (6.5)	11 (5.5)	11 (7.3)	7 (4.7)	86 (7.8)
Not classifiable	7 (3.5)	5 (2.5)	3 (1.5)	5 (2.5)	4 (2.7)	3 (2)	27 (2.5)

### Content Classification

Of the 2518 items (excluding public health–related items), a total of 2228 (88.5%) were assigned to one of 4 content categories (Table 3). The remaining 290 (11.5%) items were classified as “not classifiable” and excluded from further statistical analysis. Pathophysiology was the most frequently tested category, appearing in 981 (44%) of all classified items, followed by primary emergency care (562, 25.2%), clinical reasoning (539, 24.2%), and continued care was the least common (146, 6.6%). However, the proportion of pathophysiology items exhibited substantial fluctuations, ranging from 54.4% (240/441) in 2005 to 27.7% (78/282) in 2018. Over 23 years, the overall proportion of pathophysiology items declined significantly, from 51.6% (237/459) to 33.4% (98/293; *P*<.001). Conversely, the proportions of clinical reasoning, primary emergency care, and continued care items showed significant upward trends. From 2001 to 2024, the proportions increased from 21.6% (99/459) to 26.3% (77/293) for clinical reasoning (*P*=.01), 20.7% (95/459) to 28.7% (84/293) for primary emergency care (*P*<.001), and 6.1% (28/459) to 11.6% (34/293) for continued care (*P*<.001; Figure 3B). These trends indicate a progressive shift toward assessing clinical decision-making and patient management skills.

**Table 3:** Content classification.

Year	2001 (n=488), n (%)	2005 (n=473), n (5)	2009 (n=442), n (%)	2013 (n=436), n (%)	2018 (n=343), n (%)	2024 (n=336), n (%)	Total (n=2518), n (%)
Pathophysiology	237 (48.6)	240 (50.7)	200 (45.2)	128 (29.4)	78 (22.7)	98 (29.2)	981 (39)
Clinical reasoning	99 (20.3)	95 (20.1)	98 (22.2)	88 (20.2)	82 (23.9)	77 (22.9)	539 (21.4)
Primary emergency care	95 (19.5)	89 (18.8)	89 (20.1)	115 (26.4)	90 (26.2)	84 (25)	562 (22.3)
Continued care	28 (5.7)	17 (3.6)	17 (3.8)	18 (4.1)	32 (9.3)	34 (10.1)	146 (5.8)
Not classifiable	29 (5.9)	32 (6.8)	38 (8.6)	87 (20)	61 (17.8)	43 (12.8)	290 (11.5)

### Taxonomy Classification

All 2880 items were successfully classified into one of the 4 taxonomy types (Table 4). Type I items were the most common, accounting for 1212 (42.1%) of all items, followed by Type IIIa (n=910, 31.6%), Type II (n=541, 18.8%), and Type IIIb (n=217, 7.5%). Over 23 years, the proportion of Type I items declined significantly, from 50.7% (279/550) in 2001 to 29.5% (118/400) in 2024 (*P*<.001). Conversely, the proportions of Types II and IIIb increased significantly, from 15.1% (83/550) to 25.8% (103/400; *P*=.007) and from 3.8% (21/550) to 18.8% (75/400; *P*<.001), respectively (Figures 3C and D). Notably, the combined proportion of Types IIIa and IIIb surpassed Type I in 2018 and 2024, reflecting a progressive shift toward assessing higher-order problem-solving skills.

**Table 4:** Taxonomy classification.

Year	2001 (n=550), n (%)	2005 (n=530), n (%)	2009 (n=500), n (%)	2013 (n=500), n (%)	2018 (n=400), n (%)	2024 (n=400), n (%)	Total (n=2880), n (%)
Type I	279 (50.7)	224 (42.3)	225 (45.0)	222 (44.4)	144 (36)	118 (29.5)	1212 (42.1)
Type II	83 (15.1)	107 (20.2)	97 (19.4)	80 (16)	71 (17.8)	103 (25.8)	541 (18.8)
Type III	188 (34.2)	199 (37.6)	178 (35.6)	198 (39.6)	185 (46.3)	179 (44.8)	1127 (39.1)
Type IIIa	167 (30.4)	178 (33.6)	138 (27.6)	178 (35.6)	145 (36.3)	104 (26)	910 (31.6)
Type IIIb	21 (3.8)	21 (4)	40 (8)	20 (4)	40 (10)	75 (18.8)	217 (7.5)

### Topic Modeling

**Figure 4:**
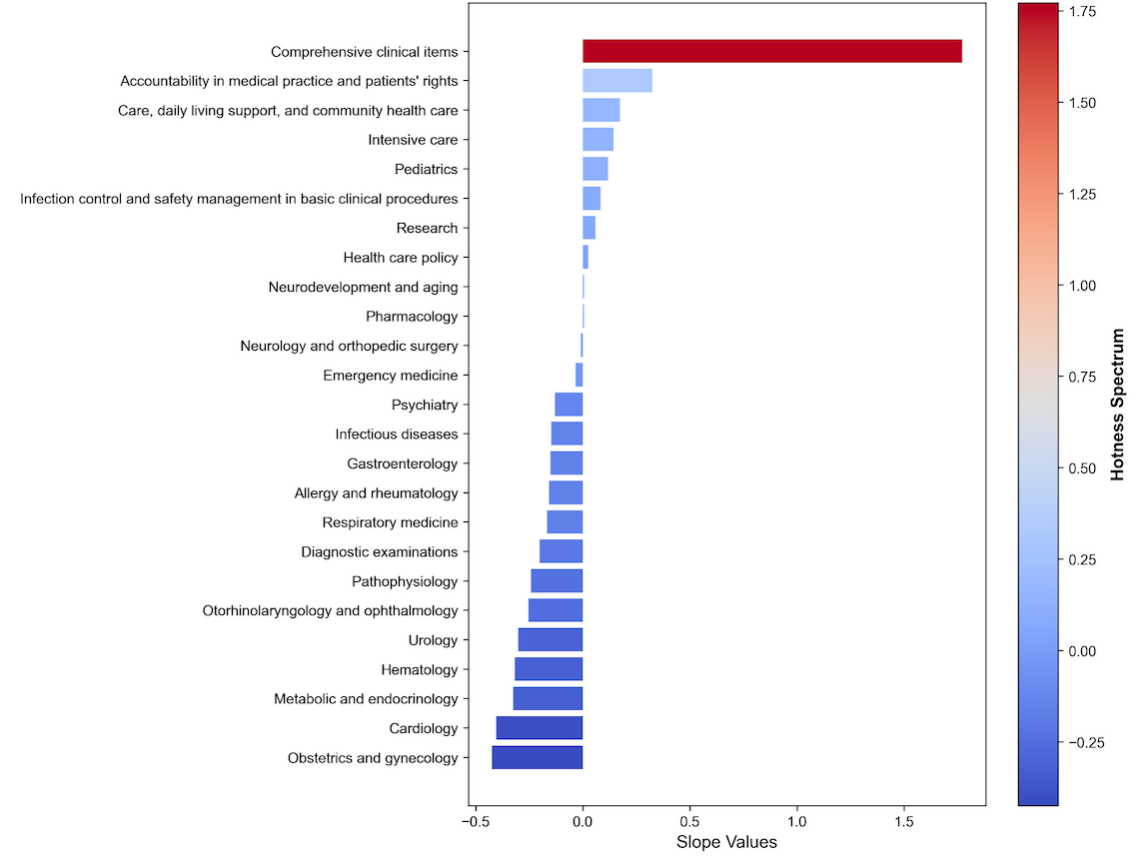
Hot and cold topics. Bar chart showing topic trends from 2001 to 2024, based on the slope of linear regression lines fitted to topic proportions over time. Topics are sorted in descending order of slope, with “hot” (increasing) topics at the top and “cold” (decreasing) topics at the bottom.

Topic modeling effectively classified 10,129 of the 11,540 (88%) items into 25 topics. The remaining 1411 (12%) items were treated as outliers and could not be assigned to any topic. A summary of these topics is presented in Table 5. Further details on these topics are presented in Multimedia Appendix 6. The most frequently appearing topics included “comprehensive clinical items,” “pediatrics,” “accountability in medical practice and patients’ rights,” “cardiology,” and “metabolic and endocrinology.” A visualization of the popular topics is shown in Figure 4. Ten topics exhibited an increasing trend, among which topics not limited to specific organs were identified, including “comprehensive clinical items,” “accountability in medical practice and patients’ rights,” “care, daily living support, and community health care,” “intensive care,” and “infection control and safety management in basic clinical procedures.”

**Table 5:** Summary of the topics.

No	Topic name	Number of items, n (%)
1	Comprehensive clinical items	4515 (39.12)
2	Pediatrics	752 (6.52)
3	Accountability in medical practice and patients’ rights	412 (3.57)
4	Cardiology	381 (3.30)
5	Metabolic and endocrinology	324 (2.81)
6	Obstetrics and gynecology	305 (2.64)
7	Diagnostic examinations	303 (2.63)
8	Otorhinolaryngology and ophthalmology	288 (2.50)
9	Emergency medicine	271 (2.35)
10	Hematology	239 (2.07)
11	Pathophysiology	237 (2.05)
12	Neurology and orthopedic surgery	208 (1.80)
13	Respiratory medicine	208 (1.80)
14	Urology	190 (1.65)
15	Care, daily living support, and community health care	183 (1.59)
16	Infectious diseases	171 (1.48)
17	Research	171 (1.48)
18	Neurodevelopment and aging	169 (1.46)
19	Psychiatry	154 (1.33)
20	Gastroenterology	147 (1.27)
21	Allergy and rheumatology	139 (1.20)
22	Pharmacology	128 (1.11)
23	Intensive care	122 (1.06)
24	Health care policy	60 (0.52)
25	Infection control and safety management in basic clinical procedures	52 (0.45)

## Discussion

### Principal Results

This study revealed significant changes in the Japanese NMLE over the past 2 decades, highlighting a shift in content and cognitive complexity. The increasing emphasis on common diseases (level A), with a corresponding reduction in highly specialized or rare conditions, aligns with the NMLE Content Guidelines’ focus on core medical knowledge relevant to general clinical practice [9]. Additionally, the dominance of pathophysiology-related items decreased. Concurrently, clinical reasoning, primary emergency care, and continued care have become more prominent, reflecting a transition from knowledge-based assessment to practical clinical skills and decision-making. Furthermore, the decline in factual recall items (Type I) and rise in higher-order cognitive skill items (Type IIIb), which require data interpretation and problem-solving, indicate a broader educational shift toward competency-based medical education, consistent with the MCC’s emphasis on clinical reasoning and problem-solving abilities. These findings suggest that the NMLE is evolving into a more practice-oriented examination, better aligned with the skills required for real-world clinical settings, including clinical clerkship.

The level classification analysis indicates that common diseases (level A) have been increasingly emphasized in the NMLE, with their proportion rising significantly in recent years. This suggests a greater focus on common diseases, while highly specialized or less frequently encountered conditions have been increasingly restricted in the examination content. This shift aligns with the policies outlined in the NMLE Content Guidelines, which emphasize core medical knowledge applicable to general clinical practice. Although the level classification was first formally introduced in the 2024 edition of the NMLE Content Guidelines, efforts to minimize discrepancies between the MCC and NMLE Content Guidelines by aligning lists of examinable diseases have already been reported [13]. To fully integrate this shift in medical education, students must be sufficiently exposed to common diseases during clinical clerkships [29,30]. Since university hospitals often manage complex or rare cases [31], structured rotations in regional or community hospitals, where students can encounter a broader range of common conditions, may enhance their preparedness for the NMLE and future clinical practice. In this context, future analyses of the NMLE based on level classification will be essential for monitoring the alignment between national medical education policies, clinical clerkship experiences, and licensing examinations.

The results of the content classification analysis indicated a decreasing dominance of pathophysiology-related items, with a concurrent increase in items assessing clinical reasoning, primary emergency care, and continued care. This suggests a transition from a knowledge-based focus to a more practice-oriented approach in the NMLE, emphasizing clinical decision-making and patient management skills. Compared with pathophysiology, primary emergency care and continued care require more practical competence, as they involve real-time decision making, prioritization, and the ability to manage ongoing patient care [32,33]. Such changes align with the directive of the NMLE Content Guidelines to assess the practical knowledge and skills acquired through clinical clerkship. Moreover, this shift is consistent with the ongoing transition in Japanese medical education, from a discipline-based to a competency-based framework, as promoted by the MCC. Notably, the NMLE blueprint also specifies organ system-based distributions (eg, nephrology, urology, and reproductive medicine: approximately 12%), and these proportions have remained relatively stable across the years. Because such distributions are explicitly monitored and reported in the blueprint, further analysis of organ system–based proportions would provide limited additional value. Conversely, the categorization into pathophysiology, clinical reasoning, primary emergency care, and continued care was newly introduced in the 2024 (Reiwa 6) NMLE Content Guidelines, without any specified proportions. Therefore, retrospectively applying this framework to past examinations enabled us to conduct the first longitudinal analysis of how the examination’s process-oriented emphases have evolved. This approach provides educators and policymakers with unique insights that complement the existing organ system-based blueprint.

Taxonomy classification analysis highlights a declining emphasis on simple factual recall items (Type I), with a corresponding increase in items requiring data interpretation and a critical analysis of answer choices (Types II and III). Notably, the proportion of Type IIIb items requiring interpretation of both the stem and answer choices increased. This suggests that the NMLE’s emphasis on higher-order cognitive skills extends beyond surface-level modifications in question format, reflecting a genuine shift toward assessing problem-solving and clinical reasoning abilities. This trend can be understood from the perspective of Revised Bloom’s Taxonomy, which categorizes cognitive processes into 6 levels: remembering, understanding, applying, analyzing, evaluating, and creating [20,21,34]. While traditional medical assessments have focused primarily on recall (remembering) and comprehension (understanding), the increasing prevalence of Type IIIb items suggests a shift toward the “analyzing” level, where examinees must break down complex clinical information, assess relationships between data points, and apply their knowledge in a more integrative manner. These changes align with the MCC’s competency framework, particularly in the problem-solving domain, which aims to develop medical graduates capable of integrating evidence-based medicine, experiential learning, and clinical reasoning to address complex patient issues. Consequently, the NMLE increasingly demands the ability to apply knowledge and critically evaluate and interpret clinical scenarios, requiring a higher degree of practical competence.

### Educational and Assessment Implications of the Distinction Between Types IIIa and IIIb

Our introduction of Type IIIa (interpretation + recall-based) and Type IIIb (interpretation + patient-specific contextualization) further clarifies the trend toward practical competence. The increasing proportion of Type IIIb items highlights the NMLE’s emphasis on context-sensitive clinical decision-making, where even after reaching a diagnosis, learners must make management choices based on severity, comorbidities, contraindications, or patient-specific factors. From an educational perspective, this has several implications. First, students can be guided away from “one disease–one answer” memorization and toward comparative reasoning among multiple plausible options. The level classification can help prioritize high-yield diseases for such practice. Second, curricula should incorporate structured case-based or simulation-based exercises that vary clinical modifiers (eg, renal function, allergies, and social context), requiring explicit justification of choices. Third, the use of visual or video-based materials may further reinforce the reasoning processes captured by Type IIIb, as they allow learners to engage with patient cues and procedural nuances beyond what text alone can convey [35]. For examination development, the IIIa-IIIb framework offers guidance for constructing items that embed contextual modifiers, use distractors requiring applied reasoning, or leverage multimedia formats. Collectively, these strategies can foster more authentic assessment and deeper learner preparation.

The following section discusses selected topics that show an increasing trend in the topic-modeling analysis.

- Comprehensive clinical items: Approximately 40% of the items are categorized under this topic, which includes questions that ask examinees to select appropriate diagnoses, tests, or treatments based on a patient’s history, physical findings, and test results, excluding discipline- or organ-specific cases. As many items from various departments share a common format, they appear to have been grouped into this overarching category. For examinees, these items require a cross-disciplinary perspective because the relevant organ system is not immediately apparent at first glance. The increasing prevalence of such items likely reflects the shift in medical education toward a competency-based approach, moving away from traditional discipline-based outcome assessments [36].
- Accountability and patients’ rights: This topic pertains to the knowledge essential for the prevention and management of medical accidents, as well as physicians’ accountability to patients and society, and how they confront such responsibilities. This overlaps with 2 of the 4 pillars of professionalism described by Arnold and Stern’s model: accountability and altruism [37]. These elements are considered fundamental components of medical professionalism. According to the MCC, professionalism is to “acknowledge the professional responsibility of physicians to be deeply involved in people’s lives and to protect health, respect diversity and humanity, and take an altruistic approach to medical practice throughout one’s career” [9]. Each revision of the MCC has increasingly emphasized the importance of professionalism. The current version lists it as the foremost quality and competency required by physicians. This heightened emphasis likely contributed to the observed increase in this topic.
- Care, daily living support, and community health care: This topic encompasses items related to long-term care insurance policies, the roles of long-term care hospitals and welfare facilities, and housing with daily living support. In Japan, where the population is rapidly aging alongside a declining birthrate, the comprehensive and seamless provision of health care, long-term care, and social support services within the community has become increasingly important [38]. This growing need is reflected in the increased attention given to this topic following the government’s introduction of a community-based integrated care system in 2006.
- Infection control and safety management in basic clinical procedures: This topic encompasses items that assess knowledge related to safety management and infection control during basic clinical procedures, such as the safe implementation of blood collection and intravenous access, appropriate handling of collected specimens, hand hygiene, and precautions against needlestick injuries. While such clinical skills are also evaluated in the objective structured clinical examination (OSCE), concerns have been raised regarding the lack of sufficiently validated OSCE stations for assessing patient safety competencies [39]. Medical incidents began to attract public attention in the 2000s, culminating in the establishment of a Medical Accident Investigation System in 2014. Reflecting the growing emphasis on patient safety, the 2018 revision of the examination guidelines incorporated terminology such as “medical accident investigation system,” “medical accident prevention manual,” and “medical safety management departments and risk managers.” The increase in items from this topic area on the NMLE has likely been in response to this heightened awareness.

The observed trends in the NMLE reflect the broader expectations of undergraduate medical education in Japan. There is a growing emphasis on acquiring practical knowledge that cannot be gained solely through lectures, developing problem-solving skills for real-world clinical challenges, and fostering deeper analytical thinking beyond rote memorization [40,41]. Given these trends, clinical clerkships play a crucial role in medical education [42-45]. Japanese medical schools allocate an average of 67 weeks to clinical training [1]. While efforts have been made to extend clerkship duration, the focus must shift toward ensuring that these experiences are participatory rather than observational. The MCC and Japan Accreditation Council for Medical Education advocate participatory clinical clerkship models, emphasizing active involvement in patient care [46]. Furthermore, clinical clerkships at community hospitals could be strategically utilized if the goal is to enhance exposure to common diseases [47-49]. In addition, the topic-modeling results suggest that themes showing an increasing trend, such as “care, daily living support, and community health care,” can be particularly well demonstrated and understood through community hospital clerkships, wherein students are more likely to encounter patients requiring long-term care, home-based support, and interprofessional collaboration. Recent studies have highlighted the educational value of such experiences for developing patient-centered and community-oriented care competencies [50,51]. A well-balanced integration of university-affiliated hospital rotations and regional hospital training will foster more practical hands-on learning experiences in undergraduate medical education.

In addition, the major topics highlighted in this study, such as “comprehensive clinical questions” and “accountability in medical practice and patients’ rights,” were robust to reasonable variations in parameter settings (eg, *n_neighbors, n_components,* and *min_cluster_size*). The same topics were consistently extracted across these sensitivity checks and continued to demonstrate increasing trends, suggesting that this study’s main conclusions are stable and not merely artifacts of specific parameter configurations.

### Practical and Policy Implications

The findings of this study provide practical insights for both policymakers and educators. For policymakers, longitudinal analysis of the NMLE offers an evidence-based foundation for examination reform, helping ensure a balance between theoretical knowledge and practical competencies, and between common and specialized conditions. Meanwhile, educators can apply the hybrid methodology demonstrated in this study to evaluate whether undergraduate curricula are aligned with national examination priorities and to analyze internal institutional examinations for continuous improvement of test content balance. From a curricular perspective, structuring clerkships to include rotations at community hospitals provides valuable opportunities for students to directly encounter common diseases, continuity of care, and outpatient management in real-world contexts. Such experiences complement the predominantly specialty-oriented training at university hospitals and strengthen the acquisition of the competencies emphasized by recent trends in the NMLE.

### Limitations

This study has several limitations that should be considered when interpreting the findings. First, the classification framework used in this study was designed specifically for the Japanese NMLE, based on the perspectives outlined in the NMLE Content Guidelines. Therefore, applying this methodology to other medical licensing examinations would require modifications to reflect country-specific guidelines and educational frameworks. In particular, regional variations in disease prevalence, differences in the expected competencies of first-year physicians, and the complexity of taxonomy classifications based on examination formats must be considered when adapting this approach to other national examinations. Second, while interrater reliability was generally acceptable, the κ coefficient for the content classification in specific examination sessions was lower than ideal. In particular, the 2013 examination (107th NMLE) showed a moderate κ coefficient (0.54), which was lower than that of other years. Closer examination revealed that many disagreements arose between the categories “pathophysiology” and “not classifiable.” The 2013 examination also represented a transitional phase in test design, as the proportion of “pathophysiology” items decreased markedly compared to earlier years (eg, 2009), while the proportion of “not classifiable” items increased. These shifts suggest that evolving examination policy and the emergence of intermediate item styles between pathophysiology and other categories contributed to assessor disagreement and the lower κ coefficient. To improve the classification reliability in future studies, it may be beneficial to use experienced physicians instead of clerkship students as assessors, particularly for the content classification, which requires clinical judgment. Third, approximately 11.5% of items in the content classification were categorized as “not classifiable.” This group was heterogeneous and included items such as specific cautions for diagnostic procedures, drugs, and surgical techniques; calculation problems (eg, A–aDO₂); and procedural tasks such as donning and doffing personal protective equipment. Although these are clinically relevant, they do not align neatly with disease- or patient-centered cognitive processes and, therefore, were grouped under “not classifiable” in this study. Representative examples are provided in Multimedia Appendix 4. However, because of their diversity, designing a more detailed classification system would be necessary to identify meaningful patterns, and future systematic analyses of this group may provide valuable insights for medical education and examination design. Fourth, public health–related items were excluded from the content classification analysis. While this decision was made to maintain consistency with the focus on clinical competencies, public health items can vary in their emphasis on factual knowledge versus practical applications. Future research should explore how public health–related items align with competency-based medical education frameworks, especially as global health and preventive medicine are increasingly emphasized in clinical training. Fifth, this study did not assess item difficulty levels; instead, it focused on the examination content and cognitive complexity. However, item difficulty plays a crucial role in examinations wherein the absolute grading criteria determine the pass–fail outcomes. If these findings are to be applied to other examinations, the potential confounding effect of difficulty adjustments must be considered, as difficulty regulation could influence the trends observed in this study. Sixth, this study did not consider the impact of examination preparation on the taxonomy classification. Because Japanese medical students frequently study past NMLE items, previously used or closely resembling past items may be answered at a lower cognitive level than initially intended. This effect is particularly relevant for examinations with a high proportion of reused or recycled items, wherein students may rely on pattern recognition rather than problem-solving skills. If this classification system is to be applied to such examinations, additional considerations in taxonomy categorization may be necessary. Finally, while BERTopic provided valuable insights by capturing contextual information more effectively than traditional topic-modeling methods such as LDA, it also introduced specific challenges when attempting a more rigorous validation. We adjusted several model parameters (Multimedia Appendix 8) to obtain a reasonable number of topics; however, the relative ranking of topics and the slopes of their temporal trends in the linear regression were sensitive to these settings. Therefore, it should be noted that the statistical robustness of these observations is not guaranteed, although our discussion focused on topics that consistently appeared at the top or showed increasing trends over time. Moreover, a key characteristic—both a potential strength and limitation—of this method is its flexibility: entirely different parameter configurations may yield novel and potentially insightful topics. Nevertheless, because the interpretation of topics ultimately relies on human judgment, there is an increased risk that certain outputs may lack clear interpretability or thematic coherence.

### Conclusions

This study identified significant shifts in the content and cognitive complexity of the Japanese NMLE over the past 2 decades. The findings indicated a greater emphasis on common diseases (level A) and a decline in highly specialized topics, suggesting a prioritization of core medical knowledge applicable to general clinical practice. Additionally, the transition from content heavy on pathophysiology to a greater focus on clinical reasoning, primary emergency care, and continued care reflects a broader shift in assessment priorities, aligning with competency-based medical education reforms in Japan.

Moreover, the reduction in factual recall items (Type I) and increase in problem-solving items (Type III), particularly those requiring dual interpretation (Type IIIb), indicate a growing emphasis on higher-order cognitive skills. These trends correspond with the MCC’s competency framework, particularly its problem-solving domain, which aims to develop physicians capable of integrating evidence-based medicine, clinical reasoning, and patient-centered care.

Topic modeling using NLP suggested an increasing emphasis on clinical items considered from a cross-organ perspective. In addition, topics not limited to specific organ systems that are increasingly represented in the NMLE, such as “accountability in medical practice and patients’ rights,” “care, daily living support, and community health care,” and “infection control and safety management in basic clinical procedures,” were identified. These trends are considered to reflect broader societal developments.

The observed changes in the NMLE suggest that Japanese undergraduate medical education is evolving to place greater importance on practical problem-solving abilities than on rote memorization. Given this trend, enhancing participatory clinical clerkship and leveraging regional hospital rotations will be crucial in further aligning medical education with evolving expectations. Future research should continue to monitor the alignment between national medical education policies and licensing examinations to ensure that assessments reflect the skills and knowledge necessary for competent medical practice.

## Appendix

Multimedia Appendix 1 English Summary of National Medical Licensing Examination (NMLE) Content Guidelines.

Multimedia Appendix 2 Mapping of the sections to subjects.

Multimedia Appendix 3 Manual for level classification.

Multimedia Appendix 4 Manual for content classification.

Multimedia Appendix 5 Manual for taxonomy classification.

Multimedia Appendix 6 Detailed topic information extracted by the topic modeling.

Multimedia Appendix 7 Interrater reliability of the classifications.

Multimedia Appendix 8 Model parameters.

## Abbreviations

AI: artificial intelligence
BERT: bidirectional encoder representations from transformers
BERTopic: bidirectional encoder representations from transformers–based topic modeling framework
c-TF-IDF: class-based term frequency–inverse document frequency
HDBSCAN: Hierarchical Density-Based Spatial Clustering of Applications with Noise
LDA: Latent Dirichlet Allocation
MCC: Model Core Curriculum
NLP: natural language processing
NMF: Non-Negative Matrix Factorization
NMLE: National Medical Licensing Examination
OSCE: objective structured clinical examination
UMAP: Uniform Manifold Approximation and Projection
USMLE: United States Medical Licensing Examination

## Data Availability

The datasets generated and analyzed in this study are available from the corresponding author upon reasonable request. However, the original datasets are owned by Medic Media Corp, Tokyo, Japan, and any use of the data requires permission from the company.
