# Supplementary material for: Trends in the Japanese National Medical Licensing Examination: Cross-Sectional Study
Source: JMIR Med Educ. 2025 Dec 23;11:e78214. doi: 10.2196/78214 (PMC12775762; doi:10.2196/78214)
Supplement: Multimedia Appendix 1 [file mededu_v11i1e78214_app1.docx]

## Supplementary file 1 - English Summary of NMLE Content Guidelines

This document provides an English summary of the NMLE Content Guidelines (“Ishi Kokka Shiken Shutsudai Naiyō Shishin (Reiwa 6 Edition)” issued by the Ministry of Health, Labour and Welfare of Japan, dated March 31, 2023.

#### 1. What are the NMLE Content Guidelines?

- Definition: The NMLE Content Guidelines summarize the appropriate scope and level of the NMLE by item and serve as the standard for examiners to follow when creating items.
- Fundamental Concepts: The overall focus is on confirming the level of achievement attained just before the start of clinical training, prioritizing the learning outcomes from clinical clerkships. The “Essential Section” primarily covers the basic clinical competence, including the fundamental professional attitude expected of a physician. “General Clinical Section” and “Specialized Clinical Section” are, in principle, limited to content that can be applied in any medical institution in Japan.
- Relation to Pre-Graduate Education, etc.: The NMLE Content Guidelines classify and specify the basic knowledge, skills, and attitudes that physicians should possess at a minimum at the point of embarking on physician duty, incorporating pre-graduate education, post-graduate clinical training, and specialty training. The Guidelines are not intended to encompass all of pre-graduate education, nor do they constrain the methods and content of pre-graduate education. They merely indicate the necessary items for fulfilling a physician's duties.

#### 2. Guide to Using the NMLE Content Guidelines

Users should follow the instructions below:

1. How to Use Examination Items and Level Classification:

- Chapters serve as headings, categorizing the principal items within both the “General Clinical Section” and “Specialized Clinical Section”.
- Domains function as headings, grouping the intermediate items.
- Sub-domains enumerate the disease concepts, classifications, or names of subjects/diseases/disorders that define the scope of the NMLE.
- Specific Items list the names of specific subjects, diseases, or disorders subsumed under the sub-domains, thereby clarifying the examination's scope to the greatest extent possible.
- For specific items situated within the “Specialized Clinical Section”, the requisite breadth of knowledge is delineated by Level Classification.

| Level Classification | | | | |
| --- | --- | --- | --- | --- |
| Level | Disease types | Competencies required for initial treatment | Competencies required for subsequent treatment | Knowledge to be tested |
| A | Common diseases in primary care settings and urgent diseases requiring emergency treatment | Possess enough knowledge to diagnose and manage patients under supervision, while appropriately consulting attending physicians as necessary | Possess enough knowledge to solve problems arising in subsequent treatment | - Knowledge of pathophysiology - Clinical reasoning skills - Knowledge of primary emergency care - Knowledge of continued care |
| B | Diseases that should be learned during postgraduate clinical training | Possess enough fundamental knowledge to manage patients under supervision | Possess enough knowledge to recognize when and how to present concerns to supervisors | - Knowledge of pathophysiology - Clinical reasoning skills - Knowledge of primary care |
| C | Diseases requiring a high level of clinical experience (beyond the postgraduate clinical training level) | Able to integrate the understanding of the outline of the illnesses and clinical reasoning to reach a differential diagnosis |  | - Ability to recall the names of diseases |

1. How to Use the Blueprint:

- The Blueprint (NMLE Design Chart) indicates the distribution ratio of items for the Chapters and Domains of the NMLE Guidelines.
- For Sub-domains and lower levels, no specific distribution ratio is given; therefore, the Examination Committee can decide on their inclusion. However, priority will be given to subjects concerning diseases and conditions frequently encountered in daily clinical practice.

#### 3. Examination Items and Level Classification

(*Some examples are shown below; others are omitted.)

Essential Section

| Domain | Sub-domain | Specific item |
| --- | --- | --- |
| 1. Professionalism in Medicine | A. Medical Ethics | 1. Ethical Issues Related to Life and Death |
|  |  | 2. Ethical Guidelines for the Medical Profession |
|  |  | 3. Beneficence, Non-maleficence, Respect for Autonomy, Justice |
| … | … | … |

General Clinical Section

| Chapter | Domain | Sub-domain | Specific item |
| --- | --- | --- | --- |
| I. Social Medicine | 1. Concepts of Health, Disease, and Disability, and the Social Environment | A. Concept of Health | 1. Definition of Health |
|  |  | B. Environment and Health | 1. Host |
|  |  |  | 2. Etiologic Agent |
| … | … | … | … |

Specialized Clinical Section

| Chapter | Domain | Sub-domain | Specific item | Level |
| --- | --- | --- | --- | --- |
| I. Congenital Anomalies, Perinatal Abnormalities, and Abnormalities of Growth and Development | 1. Abnormalities of Pregnancy | A. Abnormalities in Early Pregnancy | 1. Hyperemesis Gravidarum | A |
|  |  |  | 2. Ectopic Pregnancy | A |
|  |  |  | 3. Miscarriage and Threatened Miscarriage | A |
| … | … | … | … |  |

#### 4. Blueprint

(*Some examples are shown below; others are omitted.)

Essential Section

| Domain | Ratio |
| --- | --- |
| 1. Professionalism in Medicine | approx. 4% |
| 2. Society and Medicine | approx. 6% |
| … | … |
| 18. General Liberal Arts | approx. 2% |

General Clinical Section

| Chapter | Ratio |
| --- | --- |
| I. Social Medicine | approx. 13% |
| II. Prevention and Health Management / Promotion | approx. 17% |
| … | … |
| IX. Treatment | approx. 13% |

Specialized Clinical Section

| Chapter | Ratio |
| --- | --- |
| I. Congenital Anomalies, Perinatal Abnormalities, and Abnormalities of Growth and Development | approx. 5% |
| II. Psychiatric and Psychosomatic Disorders | approx. 5% |
| … | … |
| XIII. Disorders Caused by Environmental and Occupational Factors | approx. 5% |
