# Supplementary material for: Trends in the Japanese National Medical Licensing Examination: Cross-Sectional Study
Source: JMIR Med Educ. 2025 Dec 23;11:e78214. doi: 10.2196/78214 (PMC12775762; doi:10.2196/78214)
Supplement: Multimedia Appendix 2 [file mededu_v11i1e78214_app2.docx]

## Supplementary file 2 - Mapping of the sections to subjects

Figure S1. Approximate mapping of the sections to subjects.


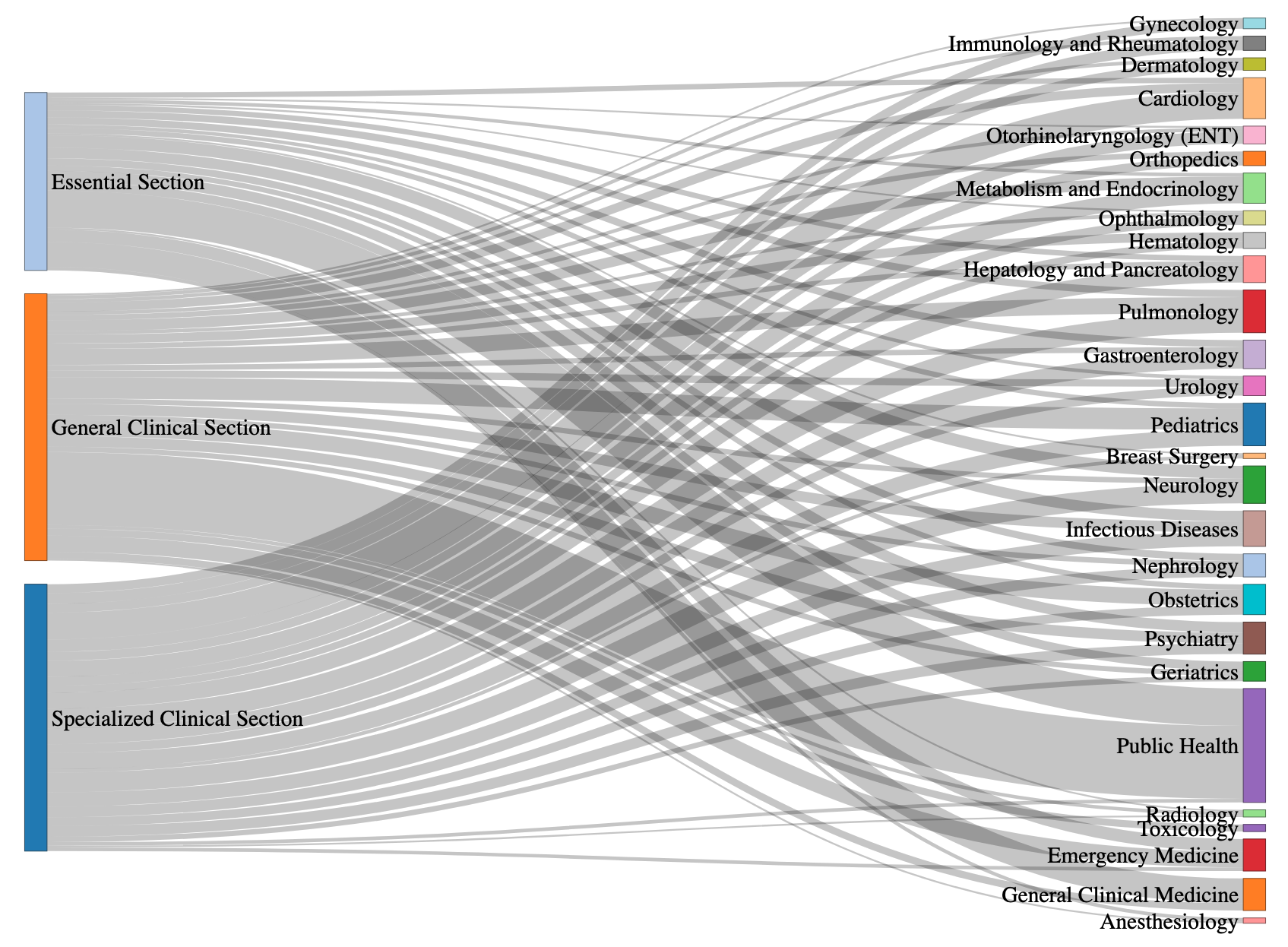


This figure is based on the 118th edition of the Question Bank (QB) for the 2024 NMLE, a widely used commercial exam review book. Since the Ministry of Health, Labor and Welfare does not publicly disclose the correspondence between sections and medical specialties, this mapping is an approximate reference derived from available resources.
