# Supplementary material for: Trends in the Japanese National Medical Licensing Examination: Cross-Sectional Study
Source: JMIR Med Educ. 2025 Dec 23;11:e78214. doi: 10.2196/78214 (PMC12775762; doi:10.2196/78214)
Supplement: Multimedia Appendix 3 [file mededu_v11i1e78214_app3.docx]

## Supplementary file 3 - Manual for Level Classification

The Level Classification of items in this study is based on the levels assigned to each item in the 2024 edition of the NMLE Content Guidelines. These guidelines designate a level—either a, b, or c—to each listed item, including specific diseases, disorders, and conditions, reflecting the expected depth and breadth of knowledge required. Guided by this framework, the classification of each item will be conducted through the following procedure:

#### Step 1: Review of the Item Text

- Carefully read the item to identify the main disease or condition being addressed.
- Record the corresponding level from the examination guidelines for the identified condition.
- If the item involves multiple key conditions (e.g., a case with significant comorbidities), record the level for each relevant condition.
- If the condition is not listed in the examination guidelines, record it as “Not Listed.”

#### Step 2: Review of the Answer Choices

- This step applies only if the answer choices consist of disease names or keywords equivalent to specific conditions.
- Identify the correct answer(s), and record their corresponding level(s) based on the examination guidelines.
- If there are multiple correct options, record the level for each.
- If a correct option is not listed in the examination guidelines, record it as “Not Listed.”

#### Step 3: Determination of Item Level

- Determine the overall level of the item based on the information gathered in Steps 1 and 2.
- Compare the levels recorded in Step 1 (selecting the highest level* if multiple are present) and those from Step 2 (again, selecting the highest level* if multiple).
- *When determining the “highest” level, the following hierarchy is applied: a > b > c.
- If the levels from Step 1 and Step 2 match, this level is assigned to the item.
- If the levels differ, prioritize the level corresponding to the condition that best represents the core focus of the item.
