# Supplementary material for: Trends in the Japanese National Medical Licensing Examination: Cross-Sectional Study
Source: JMIR Med Educ. 2025 Dec 23;11:e78214. doi: 10.2196/78214 (PMC12775762; doi:10.2196/78214)
Supplement: Multimedia Appendix 4 [file mededu_v11i1e78214_app4.docx]

## Supplementary file 4 - Manual for Content Classification

The Content Classification of items in this study is derived from the “Knowledge to be tested” categories presented in the table of the 2024 edition of the NMLE Content Guidelines [Table 1]. Each content label has been defined based on the specific aspect of clinical thinking processes that the item aims to assess in the examinee. The correspondence between label names and classification criteria is shown in the table below.

| Table S1. Criteria of Content Classification | | | |
| --- | --- | --- | --- |
| Labels in this study | Conventional Categorization | The way the items are asked in the actual exams | Types of thinking processes required |
| Pathophysiology | Basic Medicine | “Which of the following findings is recognized?”, “Which of the following is true regarding the condition?”, “Which symptoms could occur?” | Approach to thinking starting from diagnosis: drawing on and expanding relevant knowledge |
| Clinical Reasoning | Diagnosis | “Which is the diagnosis?”, “Which is the cause?”, “Which test is needed for the diagnosis?” | Approach to diagnostic thinking: narrowing down possibilities logically and identifying the diagnosis |
| Primary Emergency Care | Examintaion & Treatment | “Which is the appropriate response?”, “Which medication should be used?”, “Which test should be performed next?” | Practical approach to initial treatment: solving immediate and pressing issues |
| Continued Care | Examintaion & Treatment | “Which is the appropriate response?” (in situations such as inpatient care or regular check-up scenarios) | Practical approach to continuous treatment: anticipating and solving long-term potential issues |
| Not classifiable | - | - | Knowledge of statistics, epidemiology, knowledge specific to drugs and diagnostic methods, examination techniques, calculation problems, etc. |

- For the sake of practicality, items pertaining to normal anatomy or normal developmental processes in pediatrics are categorized under “Pathophysiology.”
- The process of “Diagnostic Recall” (i.e., recalling the name of a disease based on clinical presentation) is considered a component of “Clinical Reasoning” and is therefore not treated as a separate category but subsumed within it.
- While the term “Clinical Reasoning” is typically used in a broad sense, in the present classification it is used with a narrower focus, limited to the diagnostic aspect of clinical reasoning.

Examples corresponding to each content classification label are provided below.

| Item example 1 (Pathophysiology) | |
| --- | --- |
| A 61-year-old man visited the hospital with a chief complaint of right-sided chest pain. He had been experiencing chest pain for three months, but the pain worsened a week ago, prompting him to seek medical attention.  His vital signs were as follows: temperature 36.9°C, pulse rate 84/min regular; blood pressure 132/80 mmHg, respiratory rate 16/min, and SpO₂ 95% (room air). No abnormalities were detected in heart sounds, but decreased breath sounds were noted on the right side. Laboratory findings: Red blood cell count: 4.71 million/μL; Hemoglobin: 11.0 g/dL; Hematocrit: 36%; White blood cell count: 9,200/μL; Platelet count: 580,000/μL. Blood biochemistry: SCC: 0.7 ng/mL (reference range: ≤1.5); ProGRP: 23.8 pg/mL (reference range: ≤81); CRP: 17 mg/dL. Pleural biopsy revealed malignant cells positive for calretinin immunohistochemical staining. Chest X-ray (Figure No. 19A) and FDG-PET/CT imaging (Figure No. 19B) are provided separately.  Which of the following statements about this disease is incorrect?  a. The epithelial type is the most common. b. The prognosis is poor. c. CEA levels are within the normal range. d. Hyaluronic acid levels in pleural effusion are elevated. e. The disease develops approximately five years after asbestos exposure. |  |

| Item example 2 (Clinical Reasoning) | |
| --- | --- |
| A 3-year-old boy was brought to the hospital by his parents with complaints of fever and lower limb pain. One month ago, he twisted his left foot and experienced pain. Later, he also complained of pain in his right lower limb. Two weeks ago, he developed a fever of around 38°C, and the pain in both lower limbs worsened. He visited his primary care physician and was prescribed oral antibiotics, but the fever persisted. His physical examination revealed the following: height 103 cm, weight 17 kg, temperature 37.5°C, pulse rate 128/min regular, and blood pressure 106/70 mmHg. Several purpura were observed on both lower legs. The palpebral conjunctiva appeared pale, but no abnormalities were found in the bulbar conjunctiva. No redness was observed in the pharynx. Heart and breath sounds were normal. The abdomen was flat and soft, with no palpable liver or spleen. No superficial lymph nodes were palpable. There was no joint swelling or restriction of range of motion in the lower limbs. Laboratory findings:  Blood tests: RBC 2.98 million/μL, Hb 7.2 g/dL, Ht 23%, reticulocyte count 1.8%, WBC 15,400/μL (band neutrophils 3%, segmented neutrophils 8%, eosinophils 1%, monocytes 4%, lymphocytes 84%), platelet count 20,000/μL. Blood biochemistry: Total protein 7.5 g/dL, albumin 4.4 g/dL, total bilirubin 0.3 mg/dL, direct bilirubin 0.1 mg/dL, AST 45 U/L, ALT 19 U/L, LDH 520 U/L (reference: 190–365), ALP 180 U/L (reference: 115–359), CK 60 U/L (reference: 43–270), BUN 10 mg/dL, creatinine 0.3 mg/dL, uric acid 6.2 mg/dL, Na 140 mEq/L, K 4.0 mEq/L, Cl 101 mEq/L, Ca 11.0 mg/dL, P 6.0 mg/dL, CRP 1.2 mg/dL. X-rays of both lower limbs showed no abnormalities. A May-Giemsa-stained bone marrow smear (Figure No. 1) is provided separately.  Which of the following diseases is the most likely diagnosis?  a. Osteomyelitis b. Osteosarcoma c. Acute leukemia d. Aplastic anemia e. Hemophagocytic lymphohistiocytosis |  |

| Item example 3 (Primary Emergency Care) | |
| --- | --- |
| A 20-year-old man visited the hospital with complaints of chest discomfort and dyspnea. He had previously experienced chest discomfort several times but had never sought medical attention. Since yesterday, he developed persistent dyspnea, which prompted his visit. His past medical history was unremarkable. However, his father had a history of cerebral venous sinus thrombosis in his 20s. The patient was alert and conscious. His vital signs were as follows: temperature 36.2°C, pulse rate 96/min regular, blood pressure 104/68 mmHg, respiratory rate 24/min, and SpO₂ 94% (room air). No abnormalities were detected in heart or breath sounds. The abdomen was flat and soft, with no palpable liver or spleen. Mild edema was noted in the lower limbs. Laboratory findings: RBC 4.50 million/μL, Hb 14.5 g/dL, Ht 42%, WBC 6,200/μL (neutrophils 62%, eosinophils 1%, monocytes 5%, lymphocytes 32%), platelet count 220,000/μL. Coagulation tests: PT-INR 1.0 (reference: 0.9–1.1), APTT 30 sec (control: 32.2 sec), plasma fibrinogen 288 mg/dL (reference: 186–355), D-dimer 10 μg/mL (reference: ≤1.0). Blood biochemistry: Total bilirubin 1.1 mg/dL, LDH 208 U/L (reference: 124–222), BUN 22 mg/dL, creatinine 0.6 mg/dL, CRP 0.3 mg/dL.  Chest X-ray showed no abnormalities.  Which of the following tests is appropriate to perform immediately?  a. Bronchoscopy b. Pulmonary function test c. Neck ultrasound scan d. Chest-to-lower limb contrast-enhanced CT e. Ankle-brachial index (ABI) |  |

| Item example 4 (Continued Care) | |
| --- | --- |
| An 86-year-old man visited the hospital with a chief complaint of chest discomfort. He experienced discomfort while taking medication after dinner, which persisted and did not improve, prompting his visit. He is currently undergoing oral treatment for hypertension, dyslipidemia, hyperuricemia, benign prostatic hyperplasia, low back pain due to spinal stenosis, insomnia, and gastroesophageal reflux disease. He is scheduled to undergo cataract surgery in both eyes. He lives alone and manages his daily activities independently.  His consciousness is clear. His vital signs were as follows: height 160 cm, weight 50 kg, body temperature 36.6°C, pulse rate 80/min (regular), blood pressure 146/88 mmHg, respiratory rate 14/min, SpO₂ 98% (room air). No abnormalities were observed in the palpebral and bulbar conjunctivae. No cervical lymph nodes were palpable. Heart and breath sounds were normal. The abdomen was flat and soft, with no tenderness. No abnormalities were noted in bowel sounds.  Upper gastrointestinal endoscopy revealed a foreign body in the esophagus, which was subsequently dropped into the stomach and retrieved using forceps. The image of the gastric body from the upper gastrointestinal endoscopy (Figure No. 15) is provided separately.  Which of the following actions is appropriate to prevent recurrence?  a. Gastrostomy tube placement b. Nasogastric tube insertion c. Simplifying the prescription medication into single-dose packaging d. Switching from oral medication to injectable medication e. Separating each tablet or capsule from the PTP (blister pack) individually |  |

Item example 5 (Not classifiable)

| Which of the following is incorrect regarding the procedure for securing peripheral venous access with an indwelling needle?  a. The skin should be disinfected before puncture. b. Puncture the skin surface at an angle of 15 to 30 degrees. c. After confirming blood flow, advance the inner needle (stylet) and the catheter (outer cannula) slightly. d. Remove the tourniquet before withdrawing the inner needle. e. Recap the withdrawn inner needle. |  |
| --- | --- |

Item example 6 (Not classifiable)

| The following are the arterial blood gas (ABG) data (on room air) for a certain patient.  pH 7.40, PaCO_2_ 36 Torr, PaO_2_ 79 Torr.  Calculate the Alveolar-arterial oxygen partial pressure difference (A-aDO_2_). |
| --- |
