# Supplementary material for: Trends in the Japanese National Medical Licensing Examination: Cross-Sectional Study
Source: JMIR Med Educ. 2025 Dec 23;11:e78214. doi: 10.2196/78214 (PMC12775762; doi:10.2196/78214)
Supplement: Multimedia Appendix 5 [file mededu_v11i1e78214_app5.docx]

## Supplementary file 5 - Manual for Taxonomy Classification

This classification framework extends the taxonomy of item types presented in the official manual for the NMLE item creation by incorporating two additional categories—Type IIIa and Type IIIb—proposed specifically for this study. For each item, classification is carried out in the following sequence: first, the item stem is evaluated to determine whether interpretive processing is required; second, the answer choices are assessed to determine whether recall or interpretation is necessary. Based on these evaluations, the item is categorized as Type I, Type II, or Type III. If an item is identified as Type III, a final step is undertaken to distinguish between Type IIIa and Type IIIb, based on whether comparative judgment among the options is required.

#### Step 1. Evaluation of the Item Stem

The necessity for interpretive processing within the item stem is assessed based on the following criteria:

- Length of the item stem (word count)
- Presence of graphs that require interpretation
- Presence of images necessitating visual diagnosis
- Volume of clinical information (e.g., physical findings, vital signs, laboratory data)
- Complexity of the patient history
- Presence of clinically significant past medical history, comorbidities, or allergies

#### Step 2. Evaluation of the Answer Choices

The necessity for recall and/or interpretive processing within the answer choices is evaluated based on the following criteria:

- Whether the choices directly state the diagnosis or pathological condition derived from the item stem
- Whether the choices represent concepts that are keyword-associated with the diagnosis or condition
- Whether the choices reflect general or textbook-level knowledge regarding the diagnosis or condition
- Whether the choices are phrased in a way that demands careful reading or nuanced interpretation
- Whether the choices include images that require visual analysis or interpretation

#### Step 3. Differentiation Between Type IIIa and Type IIIb

For items classified as Type III, further differentiation is made by examining whether there are multiple answer choices that could reasonably be considered correct, depending on how broadly one interprets the clinical scenario. Type IIIb items are those in which additional reasoning is required to identify the single most appropriate answer—this may involve tailoring the response to the specifics of the clinical case presented or comparing multiple plausible choices to determine the most suitable option. In contrast, Type IIIa items do not require such comparative judgment among the choices.
