# Supplementary material for: Trends in the Japanese National Medical Licensing Examination: Cross-Sectional Study
Source: JMIR Med Educ. 2025 Dec 23;11:e78214. doi: 10.2196/78214 (PMC12775762; doi:10.2196/78214)
Supplement: Multimedia Appendix 6 [file mededu_v11i1e78214_app6.docx]

## Supplementary file 6 - Detailed topic information extracted by the topic modeling

| Table S2. Details of the topics | | |
| --- | --- | --- |
| No. | Topic Name | Key Words |
| 1 | Comprehensive Clinical Items | ['Findings', 'Visit (to hospital)', 'Criteria', 'Blood cells', 'Blood pressure', 'Male', 'Abnormality', 'Blood', 'Chief complaint', 'Respiration', 'Female', 'Pulse', 'Abdomen', 'Examination', 'Chemistry', 'Administration (of medication)', 'Consciousness', 'Chest', 'Body temperature', 'Protein'] |
| 2 | Pediatrics | ['Birth', 'Respiration', 'Male infant', 'Body weight', 'Postnatal', 'Visit', 'Fever', 'Months (old)', 'Gestational age', 'Female infant', 'Mother', 'Accompanied by', 'Findings', 'Blood cells', 'Body temperature', 'Palpable', 'Vomiting', 'Chief complaint', 'Abdomen', 'Neonate'] |
| 3 | Accountability in Medical Practice and Patients’ Rights | ['Medical care', 'Patient', 'Doctor', 'Explanation', 'Survey', 'Public health', 'Research', 'Obligation', 'Target', 'Medical examination', 'Health', 'Mother and child', 'Conduct', 'Team', 'Information', 'Necessary', 'Treatment', 'Regulation', 'Attitude', 'Insurance'] |
| 4 | Cardiology | ['Artery', 'Stenosis (narrowing)', 'Myocardium (heart muscle)', 'Closure', 'Failure', 'Mitral (valve)', 'Infarction', 'Left ventricle', 'Vein', 'Ventricle', 'Murmur', 'Atrioventricular', 'Acute', 'Atrium', 'Septum', 'Block', 'Syndrome', 'Dilation', 'Contraction', 'Angina'] |
| 5 | Metabolic and Endocrinology | ['Potassium', 'Thyroid', 'Intake', 'Calcium', 'Serum', 'Function', 'Secretion', 'High level', 'Less than', 'Syndrome', 'Sodium', 'Protein', 'Decrease', 'Inhibition', 'Aldosterone', 'Blood glucose', 'Angiotensin', 'Energy', 'Enzyme', 'Cell'] |
| 6 | Obstetrics and Gynecology | ['Pregnancy', 'Fetus', 'Amniotic fluid', 'Uterus', 'Placenta', 'Failure', 'Cell', 'Cervix', 'Concentration', 'Syndrome', 'Examination', 'Umbilical cord', 'Transplant', 'Jaundice', 'Testis', 'Function', 'In blood', 'Maternal body', 'Secretion', 'Hematopoiesis'] |
| 7 | Diagnostic Examinations | ['Artery', 'Examination', 'Head', 'CT', 'Imaging', 'Plain', 'Contrast', 'Course', 'Abdomen', 'Iliac', 'Clavicle', 'Branch', 'Conduct', 'MRI', 'Vein', 'Useful', 'Inspection', 'Diagnosis', 'Ultrasound', 'Blood sampling'] |
| 8 | Otorhinolaryngology and Ophthalmology | ['Hearing loss', 'Nerve', 'Examination', 'Disorder', 'Patient', 'Middle ear', 'Retina', 'Reflex', 'Otorrhea (ear discharge)', 'Syndrome', 'Headache', 'Fracture', 'Hearing ability', 'Nasal cavity', 'Doctor', 'Vestibular', 'Sign', 'Eardrum', 'Eye drops', 'Barré'] |
| 9 | Emergency Medicine | ['Administration', 'Conduct', 'Treatment', 'Endoscopy', 'Appropriate', 'Steroid', 'Patient', 'Procedure', 'Diuretic', 'Therapy', 'First', 'Infusion', 'Puncture', 'Intravenous injection', 'Cortex', 'Adrenal gland', 'Transfusion', 'Antibacterial', 'Vein', 'Catheter'] |
| 10 | Hematology | ['Syndrome', 'Leukemia', 'Immunity', 'Genetics', 'Cell', 'Lymph', 'Reduction', 'New drug', 'Failure', 'Acute', 'Blastoma', 'Anemia', 'Bone marrow', 'Chronic', 'Chain', 'Severe', 'Infection', 'Increase', 'Aqueduct', 'Decrease'] |
| 11 | Pathophysiology | ['Syndrome', 'Colon', 'Disorder', 'Prostate', 'Reduction', 'High level', 'Deficiency', 'Failure', 'Anemia', 'Thrombin', 'Artery', 'Disease', 'Obstruction', 'Resection', 'Medical care', 'Multiple', 'Paralysis', 'Cause', 'Elderly', 'Patient'] |
| 12 | Neurology and Orthopedic Surgery | ['Nerve', 'Joint', 'Site', 'Glove', 'Glossopharyngeal', 'Thigh', 'Palpation', 'Measurement', 'Vagus', 'Disorder', 'Fracture', 'Examination', 'Upper arm', 'Reflex', 'Body', 'Rupture', 'Kneecap', 'Swelling', 'Spine', 'Deep (reflex)'] |
| 13 | Respiratory medicine | ['Respiration', 'Trachea', 'Syndrome', 'Alveolus', 'Ventilation', 'Vein', 'Oxygen', 'Defense', 'Alkalosis', 'Decrease', 'Metabolism', 'Disease', 'Pathophysiology', 'Lobule', 'Compliance', 'Asthma', 'Acidosis', 'Acute', 'Presence', 'Gas'] |
| 14 | Urology | ['Stone', 'Bladder', 'Prostate', 'Metastasis', 'Tumor', 'Cause', 'Urinary tract', 'Ureter', 'Gallbladder', 'Syndrome', 'Acute', 'Cell', 'Adenocarcinoma', 'Cyst', 'Hematuria', 'Alcohol consumption', 'Puberty', 'Decrease', 'Calcium', 'Alcohol'] |
| 15 | Care, Daily Living Support, and Community Healthcare | ['Medical care', 'Nursing care', 'Support', 'Life', 'Community', 'Welfare', 'Public health', 'Disability', 'Home care', 'Function', 'Elderly', 'Society', 'Insurance', 'Care', 'Rehabilitation', 'Labor', 'Facility', 'Mental', 'Target', 'Patient'] |
| 16 | Infectious Diseases | ['Pneumonia', 'Virus', 'Coccus (spherical bacteria)', 'Rubella', 'Infection', 'Vaccine', 'Influenza', 'Cytomegalovirus', 'Chickenpox', 'Causative', 'Staphylococcus', 'Bacillus', 'Streptococcus', 'Pathogen', 'Antibody', 'Cause', 'Conjunctiva', 'Vaccination', 'Mycoplasma', 'Herpes'] |
| 17 | Research | ['Research', 'Death', 'Population', 'Sensitivity', 'Specificity', 'Age', 'Probability', 'Comparison', 'Group', 'Control', 'Calculation', 'Exposure', 'Asbestos', 'Mesothelium', 'Description', 'When', 'Statistics', 'Production', 'Case', 'Estimation'] |
| 18 | Neurodevelopment and Aging | ['Development', 'Reflex', 'Disorder', 'Evaluation', 'Months', 'Mother', 'Language', 'Delay', 'Examination', 'Rorschach', 'Autism', 'Mini', 'Onset', 'Mental', 'Male infant', 'Test', 'Can do', 'Intelligence', 'Worsening', 'Infant'] |
| 19 | Psychiatry | ['Disorder', 'Delusion', 'Sleep', 'Obsessive-compulsive', 'Emotion', 'Symptom', 'Self', 'Feature', 'Appear', 'Ego', 'Thought', 'Experience', 'REM', 'Amnesia', 'Situation', 'Hallucination', 'Artery', 'Anxiety', 'Depression', 'Depressive disorder'] |
| 20 | Gastroenterology | ['Shock', 'Colon', 'Esophagus', 'Burn', 'Ulcer', 'Acute', 'Constipation', 'Enteritis', 'Anaphylaxis', 'Sepsis', 'Convulsion', 'Bile duct', 'Complication', 'Diabetes', 'Syndrome', 'Edema', 'Golden', 'Hypersensitivity', 'Duodenum', 'Ischemia'] |
| 21 | Allergy and Rheumatology | ['Antibody', 'IL (interleukin)', 'Red blood', 'SLE (systemic lupus erythematosus)', 'Rheumatism', 'Erythematosus', 'Tuberculosis', 'Myositis', 'Sclerosis', 'Nephritis', 'Polyarthritis', 'Systemic', 'Syndrome', 'Autoimmune', 'Chronic', 'Serum', 'Joint', 'Skin', 'Glomerulus', 'Purpura'] |
| 22 | Pharmacology | ['Drug', 'Oral', 'Administration', 'Uremia', 'Steroid', 'Effect', 'Inflammation', 'Drink', 'Benzodiazepine', 'Promotion', 'Inhibition', 'Cortex', 'Adrenal gland', 'Synthesis', 'Phenylketonuria', 'Bisphosphonate', 'Narcotic', 'Treatment', 'Insulin', 'In blood'] |
| 23 | Intensive Care | ['Nerve', 'Trachea', 'Artery', 'Paralysis', 'Intubation', 'Airway', 'Insertion', 'Induction', 'Spleen', 'Conduct', 'Balloon', 'Performed', 'First', 'Respiration', 'IABP', 'Heart', 'Fluid', 'Nasally', 'Room admission', 'Therapy'] |
| 24 | Healthcare Policy | ['Prevention', 'Assistance', 'International', 'Health', 'Japan', 'Declaration', 'China', 'Government', 'America', 'WHO', 'Organization', 'Development', 'Prevention', 'Charter', 'Cooperation', 'ODA (Official Development Assistance)', 'World', 'Health', 'Promotion', 'Public health'] |
| 25 | Infection Control and Safety Management in Basic Clinical Procedures | ['Collection', 'Specimen', 'Infection', 'Recapping', 'Processing', 'Puncture', 'Disposal', 'Catheter', 'Culture', 'Disaster', 'Blood sampling', 'Blood', 'Set', 'Removal', 'Vein', 'Disinfection', 'Filter paper', 'Ceftriaxone', 'Conduct', 'Attach'] |

Integration of the topics

- Topic 1: "Comprehensive Clinical Items" was created by manually integrating items that had originally been extracted as separate topics—one concerning male patients and the other concerning female patients.
- Topic 7: "Obstetrics & Gynecology" was created by manually integrating two that had originally been extracted as separate topics. One mainly consisted of items centered on individual clinical cases, while the other comprised items that primarily assessed general pathophysiological knowledge.
- Topic 6: "Cardiology" was created by manually integrating two extracted topics. One primarily consisted of items related to ischemic heart disease, while the other was largely composed of items concerning heart failure.
- Topics 4, 11, and 14: “Otorhinolaryngology & Ophthalmology,” “Hematology,” and “Respiratory medicine” were each created by manually integrating two topics that had been automatically extracted and exhibited no clear differences in content.
