# Supplementary material for: Trends in the Japanese National Medical Licensing Examination: Cross-Sectional Study
Source: JMIR Med Educ. 2025 Dec 23;11:e78214. doi: 10.2196/78214 (PMC12775762; doi:10.2196/78214)
Supplement: Multimedia Appendix 7 [file mededu_v11i1e78214_app7.docx]

## Supplementary file 7 - Inter-rater reliability of the classifications

The inter-rater reliability was assessed for each iteration and each classification system using the κ coefficient. Fleiss' κ was adopted for the analysis. Because assessment for certain iterations was performed by varying pairs of assessors (instead of the same fixed duo), the process corresponds to a case where raters are sampled from a larger pool. Therefore, Fleiss' κ was the most appropriate measure for this evaluation. The calculated κ coefficients for all classifications are presented below.

| Table S3. Fleiss’ κ coefficient | | | | | | |
| --- | --- | --- | --- | --- | --- | --- |
| Year | 2001 | 2005 | 2009 | 2013 | 2018 | 2024 |
| Level classification | 0.86 | 0.79 | 0.77 | 0.74 | 0.85 | 0.81 |
| Content classification | 0.74 | 0.64 | 0.74 | 0.54 | 0.67 | 0.79 |
| Taxonomy classification | 0.82 | 0.71 | 0.74 | 0.65 | 0.62 | 0.69 |
