# Supplementary material for: Trends in the Japanese National Medical Licensing Examination: Cross-Sectional Study
Source: JMIR Med Educ. 2025 Dec 23;11:e78214. doi: 10.2196/78214 (PMC12775762; doi:10.2196/78214)
Supplement: Multimedia Appendix 8 [file mededu_v11i1e78214_app8.docx]

## Supplementary file 8 - Model Parameters

- UMPA: "n_neighbors":3, "n_components":10
- HDBSCAN: "min_cluster_size":20, "min_samples":5, "epsilon":0.1
- STOP WORDS for CountVectorizer: "mg", "dl", "kg", "ml","cm","mmHg", "mEq", "kcal", "HU", "IU", "歳", "です", "ます", "まし", "ましょう", "認め（る）", "示す", "起こす", "にくい", "やすい" , "なっ（た）", "考え", "つい（て）", "おけ（る）", "関する", "正しい","誤っ", "組合せ", "どれ", "選べ", "かつ", "解答", "求めよ", "こと", "から", "ごと", "なら", "なく", "なり", "対し", "ともなう","多い", "（に）よる", "この", "べき", "する", "ため", "（ら）れる", "ある", "ない", "なる", "いる", "よう", "など", "また", "これ", "それ", "より",
